# Energy transfer and trapping in *Synechococcus* WH 7803

**DOI:** 10.1007/s11120-017-0451-2

**Published:** 2017-10-13

**Authors:** Alonso M. Acuña, Claire Lemaire, Rienk van Grondelle, Bruno Robert, Ivo H. M. van Stokkum

**Affiliations:** 1 Faculty of Science, Department of Physics and Astronomy and Institute for Lasers, Life and Biophotonics, Vrije Universiteit, Amsterdam, Netherlands; 2 CEA, Institut de Biologie et de Technologies de Saclay, and CNRS, 91191 Gif/Yvette Cedex, France

**Keywords:** Excitation energy transfer, Global analysis, Light harvesting, Target analysis

## Abstract

Excitation energy transfer (EET) and trapping in *Synechococcus* WH 7803 whole cells and isolated photosystem I (PSI) complexes have been studied by time-resolved emission spectroscopy at room temperature (RT) and at 77 K. With the help of global and target analysis, the pathways of EET and the charge separation dynamics have been identified. Energy absorbed in the phycobilisome (PB) rods by the abundant phycoerythrin (PE) is funneled to phycocyanin (PC645) and from there to the core that contains allophycocyanin (APC660 and APC680). Intra-PB EET rates have been estimated to range from 11 to 68/ns. It was estimated that at RT, the terminal emitter of the phycobilisome, APC680, transfers its energy at a rate of 90/ns to PSI and at a rate of 50/ns to PSII. At 77 K, the redshifted Chl *a* states in the PSI core were heterogeneous, with maximum emission at 697 and 707 nm. In 72% of the PSI complexes, the bulk Chl *a* in equilibrium with F697 decayed with a main trapping lifetime of 39 ps.

**Electronic supplementary material:**

The online version of this article (doi:10.1007/s11120-017-0451-2) contains supplementary material, which is available to authorized users.

## Introduction

Photosynthesis is key to the conversion of solar energy to biomass. Light-harvesting antennae absorb sunlight and transfer the excitation energy ultimately to the reaction centers (RCs). The phycobilisome (PB) is the light-harvesting antenna of many cyanobacteria, red algae and glaucophytes (Adir 2005; Glazer 1984; Watanabe and Ikeuchi 2013). Light is absorbed by phycocyanobilin pigments that are covalently bound to phycobiliproteins (Glazer 1984). *Synechococcus* are marine cyanobacteria that are estimated to assimilate 8 Gt C/year, corresponding to ≈ 17% of the ocean net primary production (Flombaum et al. 2013). In *Synechococcus* WH7803 (Waterbury et al. 1986), the rods consist of three types of hexamers, which are named after the phycocyanin (PC) and phycoerythrin (PE) pigments they contain. A model for the PB structure has been proposed by Six et al. (2007). From tip to core, each rod consists of five hexamers: two PEII, two PEI and one R-PCII. In addition to PE, the PEII hexamer contains phycourobilin (PU) as well in a PE:PU ratio of 5:1 (Six et al. 2007). Six rods radiate from a core consisting of three cylinders that contain allophycocyanin (APC) pigments. This pattern of three cylinders and six rods is analogous to that of PBs of *Synechocystis* sp. PCC 6803 (Arteni et al. 2009). The spectral properties of the rod hexamers (Ong and Glazer 1987, 1991; Six et al. 2007) are summarized in Table 1.

**Table 1:** Absorption and emission maxima of the rod hexamers according to Ong and Glazer (1987, 1991) and Six et al. (2007)

Hexamer	A_max_ (nm)	F_max_ (nm)
PEII	544 (PEII), sh 498 (PU)	563
PEI	550 (PEI)	572
R-PCII	533, 554 (PE), 615 (PC)	646

*sh* shoulder

**Fig. 1:**
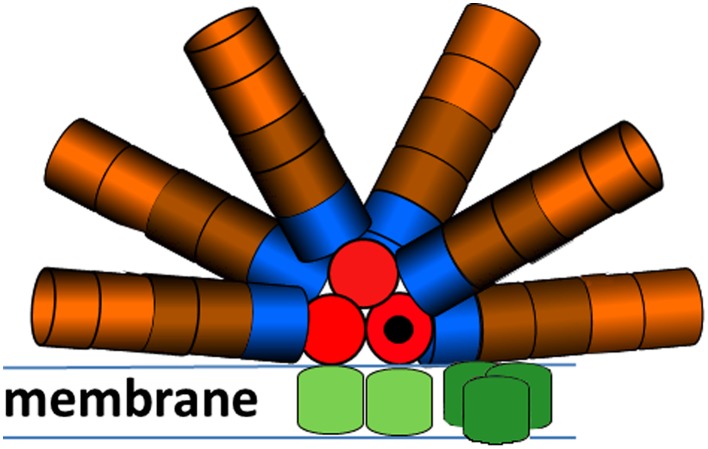
Cartoon model for the *Synechococcus* WH7803 PB structure proposed by Six et al. (2007) combined with the megacomplex structure of Liu et al. (2013). Key: PEII (brown), PEI (maroon), R-PCII (blue), APC660 (red), APC680 (black), PSII dimer (green) and PSI trimer (dark green)

Together, these PE, PC and APC pigments absorb light between 400 and 650 nm. Excitations of the PB pigments are efficiently transferred to the chlorophyll-containing photosystems (PS) I and II (Tian et al. 2011, 2012, 2013b; Scott et al. 2006; Gillbro et al. 1985; Sandstrom et al. 1988; Liu et al. 2013; Dong et al. 2009). A cartoon model for the structure of PB, assuming that PB can transfer to both PSI and PSII (Liu et al. 2013) is sketched in Fig. 1.

These photosystems convert the excitations to chemical energy via initial charge separation (van Grondelle et al. 1994), and the combined action of PBs and photosystems (the light reactions of photosynthesis) provides the energy input to the cell. Excitation energy transfer (EET) and trapping have recently been described in detail in a PSI-deficient mutant of *Synechocystis* sp. PCC 6803 (Acuña et al. 2017). By means of a target analysis (Holzwarth 1996; van Stokkum et al. 2004), it was estimated that the terminal emitter of the phycobilisome, termed APC680, transfers its energy at a rate of (20 ps)^−1^ to PSII. This is faster than the intraphycobilisome EET rates between a rod and a core cylinder, or between the core cylinders (van Stokkum et al. 2017). Here, we measure ultrafast time-resolved emission spectra in whole cells of *Synechococcus* WH7803 at room temperature and at 77 K to study EET and trapping. With the help of target analysis, we estimate the unknown rates of EET within the *Synechococcus* WH7803 PB and between the PB and the photosystems.

## Materials and Methods

### Growth conditions and sample preparation

#### Cell culture and growth conditions

*Synechococcus* sp. strain WH 7803 (from the Woods Holes Oceanographic Institution, USA) (Waterbury et al. 1986) was grown at the CEA (Saclay, FR) in an artificial seawater medium (Wyman et al. 1985) at 20 °C with 10 µmol photons m^−2^ s^−1^. The experimental cultures were continuously bubbled with sterile air.

#### Sample handling

Samples of the main batch were then taken to the LaserLab (Amsterdam, NL) and cooled in dry ice and in darkness to perform time-resolved fluorescence measurements. Before the first measurement, the cells were first acclimated for ca. 20 min to 40 µmol photons m^−2^ s^−1^ in an Erlenmeyer flask continuously shaken at 250 rpm. The flask was kept in those conditions. For each time range, a new sample was taken from the Erlenmeyer flask. Streak images were all acquired within the first 48 h after arrival of the sample at the LaserLab in Amsterdam.

#### Thylakoid extraction and PSI isolation

Thylakoid extraction was performed as described previously (Post et al. 1992). PSI was purified by sucrose density gradient centrifugation as described previously (Marquardt and Rhiel 1997).

### Steady-state absorption

Steady-state absorption of whole cells was measured using a Varian Cary 4000 UV–Vis spectrophotometer additionally equipped with a Varian 900 external diffuse reflectance accessory.

### Time-resolved fluorescence

A series of streak camera measurements (Van Stokkum et al. 2008; Wlodarczyk et al. 2016), comprising several image sequences using different parameters, was carried out less than 1 h after taking the cells out of the flask to ensure excellent sample quality. While there is no evidence for significant cell degradation during single sequences, the cells may have changed from one sequence to the next. The different conditions from one sequence to another include two different excitation wavelengths (400 nm for predominant Chl excitation; 550 nm for predominant PE excitation), or different time ranges (TR): from 0 to 400 ps (TR2) and from 0 to 1500 ps (TR4). For measurements performed using TR2 (TR4), the image sequence consists of 300 (150) images, each of which results from a scan of 8 s. To achieve a high SNR, each image sequence is used to produce an average image that is, in turn, corrected for background and lamp shading before analysis. To judge whether the sample changed over time, we kept track of the chronological order in sequence acquisition. We indicate the conditions as ‘time range/λ_exc_ (in nm)’. Measurements were done at room temperature (RT) and at 77 K. At RT, measurements were carried out with two batches A and B. In batch A, the laser power used was 45 μW and the acquisition order was: TR2/400 → TR4/400 → TR4/550 → TR2/550. In Batch B, the laser power was 60 μW and the acquisition order was: TR2/400 → TR4/400 → TR2/550 → TR4/550. At 77 K, the laser power used was 15 μW and the acquisition order was: TR4/550 → TR2/550 → TR2/400 → TR4/400. To avoid annihilation, the laser power was at most 60 μW.

The samples frozen to 77 K in a Pasteur pipette were placed in a cold finger. The optical path length within the sample was ≈ 1 mm. The fluorescence at the angle of 90° to the direction of the excitation beam was collimated and focused onto the input slit of spectrograph Chromex 250IS (Chromex, Albuquerque, New Mexico). With 550 nm excitation at RT and with both excitations at 77 K a cutoff filter OC13 was used to block the scattered excitation light. This filter attenuates light with wavelengths below 620 nm. Spectrally resolved emission was detected using a Hamamatsu C5680 synchroscan camera with a cooled Hamamatsu Digital Camera C10600-10B (ORCA-R2) (Hamamatsu Photonics, Hamamatsu, Japan). In all cases, the laser light was vertically polarized, the spot size was 60 μm, the laser repetition rate was set to 250 kHz, the input slit of the spectrograph was 140 μm and that of the photo-cathode of the streak camera was 220 μm and the detection was parallel (VV) to the incident polarization. The full width at half maximum (FWHM) of the instrument response function (IRF) was ≈ 10 ps with TR2 and ≈ 25 ps with TR4. At 77 K, The FWHM of the IRF was ≈ 17 ps with TR2 and ≈ 27 ps with TR4.

### Global and target analysis of time-resolved emission spectra

In target analysis of time-resolved emission spectra, the inverse problem is to determine the number of electronically excited states (${N_{{\text{states}}}}$) present in the system, and to estimate their spectral properties ${\text{SA}}{{\text{S}}_l}(\lambda )$ and their populations $c_{l}^{S}(t)$(superscript S stands for species). The time-resolved emission spectra ${\text{TRES}}(t,\lambda )$are described by a parameterized superposition model:

$${\text{TRES}}(t,\lambda ) = \sum\limits_{{l = 1}}^{{N_{{{\text{states}}}} }} {c_{l}^{S} (t,\theta ){\text{SAS}}_{l} (\lambda )} ,$$

where the populations are determined by an unknown compartmental model that depends upon the unknown kinetic parameters $\theta$. In the target analysis, constraints on the *SAS* are needed to estimate all parameters $\theta$ and ${\text{SA}}{{\text{S}}_l}(\lambda )$ (Snellenburg et al. 2013; van Stokkum et al. 2004).

The population of the *l*-th compartment is $c_{l}^{S}(t)$. The concentrations of all compartments are collated in a vector: $${c^S}(t)={\left[ {\begin{array}{*{20}{c}} {c_{1}^{S}(t)}&{c_{2}^{S}(t)}& \ldots &{c_{{{n_{{\text{comp}}}}}}^{S}(t)} \end{array}} \right]^T}$$ which obeys the differential equation

$$\frac{d}{{dt}}{c^S}(t)=K{c^S}(t)+j(t),$$

where the transfer matrix *K* contains off-diagonal elements ${k_{pq}}$, representing the microscopic rate constant for EET from compartment *q* to compartment *p*. The diagonal elements contain the total decay rates of each compartment. The input to the compartments is $j(t) = {\text{IRF}}(t)[ {x_{1} \ldots \;x_{{n_{{{\text{comp}}}} }} } ]^{T}$, with ${x_l}$ the absorption of the *l*-th compartment.

The impulse response of the system, which is a sum of exponential decays, has to be convolved with the IRF. Typically, a Gaussian-shaped IRF is adequate, with parameters *µ* for the location of the IRF maximum and Δ for the FWHM of the IRF:

$${\text{IRF}}(t) = \frac{1}{{\tilde{\Delta }\sqrt {2\pi } }}\exp \left( { - \log (2)(2(t - \mu )/\Delta )^{2} } \right),$$

where $\tilde {\Delta }=\Delta /(2\sqrt {2\log (2)} )$. The convolution (indicated by an *) of this IRF with an exponential decay (with decay rate *k*) yields an analytical expression which facilitates the estimation of the decay rate *k* and the IRF parameters *µ* and Δ:

$$c^{D} (t,k,\mu ,\Delta ) = \exp ( - kt){\mkern 1mu} \ast {\mkern 1mu} IRF(t) = \frac{1}{2}\exp ( - kt)\exp \left( {k\left( {\mu + \frac{{k\tilde{\Delta }^{2} }}{2}} \right)} \right)\left\{ {1 + erf\left( {\frac{{t - (\mu + k\tilde{\Delta }^{2} )}}{{\sqrt 2 \tilde{\Delta }}}} \right)} \right\}.$$

Typically, with streak camera measurements, the IRF can be well approximated by a sum of up to three Gaussians.

The solution of the general compartmental model described by the *K* matrix consists of exponential decays with decay rates equal to the eigenvalues of the *K* matrix. When the compartmental model consists of independently decaying species, their spectra are termed ${\text{DA}}{{\text{S}}_l}(\lambda )$ (decay-associated spectra), and when it consists of a sequential scheme with increasing lifetimes the spectra are termed ${\text{EA}}{{\text{S}}_l}(\lambda )$ (evolution-associated spectra).

The interrelation between the DAS and SAS is expressed in the following matrix equation:

$${C^D}(\theta ,\mu ,\Delta ) \cdot {\text{DA}}{{\text{S}}^T}={C^S}(\theta ,\mu ,\Delta ) \cdot {\text{SA}}{{\text{S}}^T}.$$

Here, the matrix ${C^D}(\theta ,\mu ,\Delta )$ contains in its *l*-th column the decay $c_{l}^{D}(t,{k_l},\mu ,\Delta )$ and the matrix ${C^S}(\theta ,\mu ,\Delta )$ contains in its columns the populations $c_{l}^{S}(t)$ of the general compartmental model.

### Simultaneous target analysis

To resolve the different species and to improve the precision of the estimated parameters, the set of ${N_{\exp }}$ experiments that describe the same sample (measured with different excitation wavelengths or on different time ranges) can be analyzed simultaneously. For each additional data set ${\text{TR}}{{\text{S}}_e}$ one scaling parameter ${\alpha _e}$ and one time shift parameter ${\mu _e}$ must be added:

$${\text{TR}}{{\text{S}}_e}={\alpha _e}({C_e}^{S}(\theta ,{\mu _e},\Delta ) \cdot {\text{SA}}{{\text{S}}^T}.$$

The different excitation wavelengths are taken into account via the absorptions of the species that result in $C_{e}^{S}(\theta ,{\mu _e},\Delta )$.

### Residual analysis

Following a successfully converged fit, the matrix of residuals is analyzed with the help of a singular value decomposition (SVD). Formally, the residual matrix can be decomposed as

$${\text{res}}(t,\lambda )=\sum\limits_{{l=1}}^{m} {u_{l}^{{{\text{res}}}}(t){s_l}w_{{_{l}}}^{{{\text{res}}}}(\lambda )} ,$$

where ${u_l}$ and ${w_l}$ are the left and right singular vectors, ${s_l}$ the sorted singular values, and *m* is the minimum of the number of rows and columns of the matrix. The singular vectors are orthogonal and provide an optimal least squares approximation of the matrix. The SVD of the matrix of residuals is useful to diagnose the shortcomings of the model used or systematic errors in the data.

## Results and discussion

### Measurements at room temperature

**Fig. 2:**
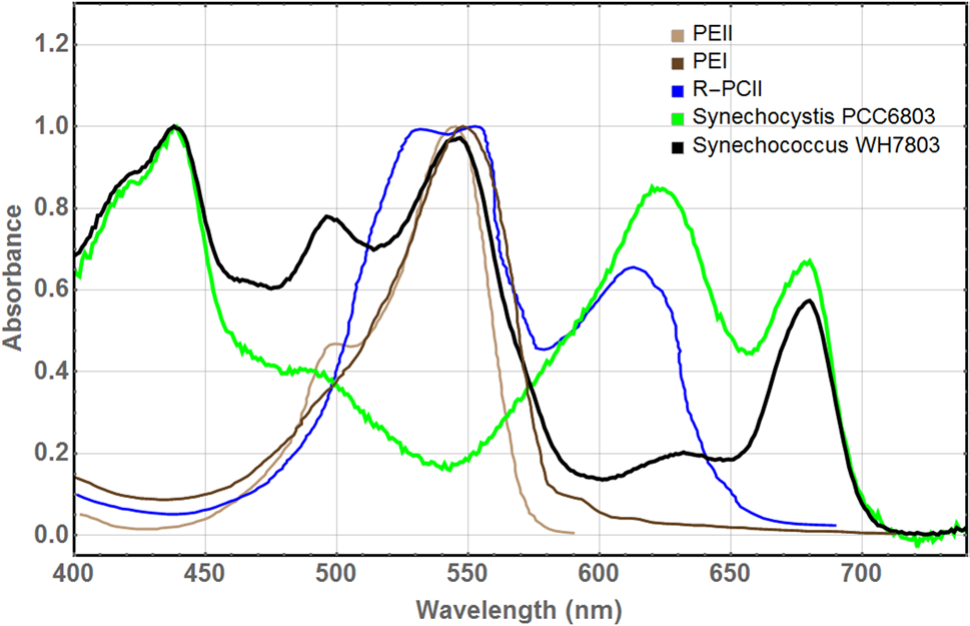
Normalized RT absorption spectra of whole cells of cyanobacterium *Synechococcus* WH7803 (black) and Synechocystis PCC6803 (green) in comparison with the spectra of PEII (maroon) and PEI (brown) complexes isolated from *Synechococcus* WH7803 (Six et al. 2007) and R-PCII (blue) in 0.05 M sodium phosphate buffer (Ong and Glazer 1987)

The RT absorption spectrum of *Synechococcus* WH7803 (black in Fig. 2) is dominated by the PE absorption around 550 nm. The absorption spectra of the three hexamers [taken from Six et al. (2007) and Ong and Glazer (1987)] are consistent with this dominant PE absorption. The absorption maximum is most close to that of PEII (brown).

**Fig. 3:**
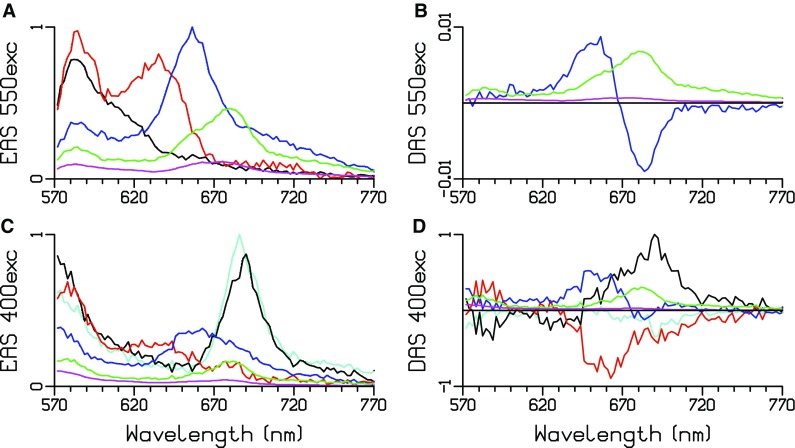
Estimated EAS and DAS after 550 (**a, b**) or 400 (**c, d**) nm excitation of whole cells of *Synechococcus* WH7803 at RT. Estimated lifetimes are collated in Table 2

At RT, a global analysis requires five or six lifetimes with 550 or 400 nm excitation, cf. Fig. 3 and Table 2. The EAS estimated with 550 nm excitation (Fig. 3a) is attenuated on the blue side because a cutoff filter had to be used to suppress the scattered excitation light. The emission below 600 nm can be attributed to PEI and PEII. The decay is multiexponential, with rise components present in the cyan and black DAS with 400 nm excitation (Fig. 3d). The first two lifetimes with 550 nm excitation are both 30 ps; thus, their DAS are compensating and cannot be plotted. A clear redshift of the PE emission is visible going from the black to the red EAS (Fig. 3a, c). After 400 nm excitation, in the Chl *a* emission region (670–700 nm), PSI equilibrates with 8.5 ps and then decays with 22 ps (cyan and black EAS in Fig. 3c). The final DAS of ≈ 1360 ps (magenta) can be interpreted as a small fraction of non-transferring PB (peaking at ≈ 677 nm) and a small fraction of non-transferring PE (peaking at ≈ 570 nm). The green DAS (≈ 130 ps) also shows two peaks (Fig. 3b, d). The largest peak (at ≈ 677 nm) can be attributed to trapping in the photosystems. The blue DAS (≈ 70 ps) is almost conservative in Fig. 3b, and can be attributed to EET from PB to the photosystems. These complicated DAS can only be further interpreted with the help of a target analysis.

**Table 2:** Estimated lifetimes (in ps) after 550 or 400 nm excitation of whole cells of *Synechococcus* WH7803 at RT

${\lambda _{'{\text{exc}}}}$ (nm)	τ1 (cyan)	τ2 (black)	τ3 (red)	τ4 (blue)	τ5 (green)	τ6 (magenta)
550	−	30	30	63	118	1374
400	8.5	22	43	79	139	1351

Our target model consists of three different complexes: a PB–PSII–PSI complex with PSII RCs open (estimated to be 79% with 550 nm excitation), non-transferring PB (14%) and non-transferring PE (7%) (Table 3).

**Table 3:** Estimated fractions of the different complexes in the experiments at RT with 400 and 550 nm excitation

	400 exc (%)	550 exc (%)
PB–PSII–PSI	80	79
Non-transferring PB	9	14
Non-transferring PE	10	7

**Fig. 4:**
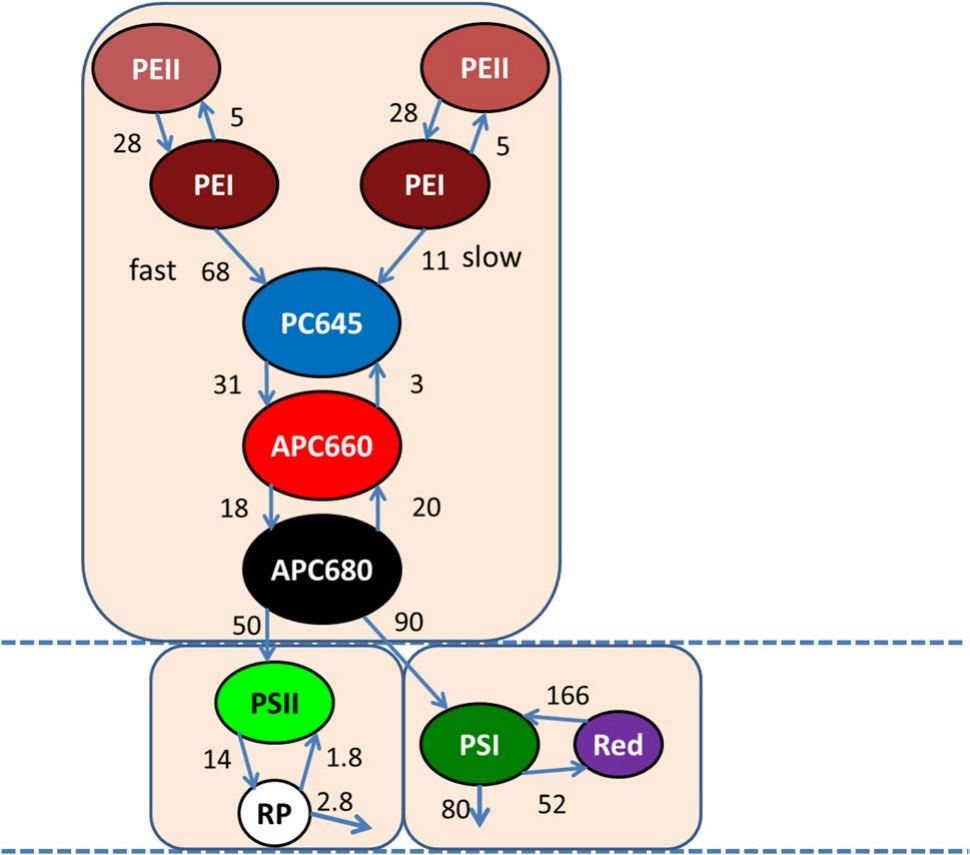
Functional compartmental model of the PB–PSII–PSI complex with RCs open at RT. All microscopic rate constants are in 1/ns. The common ${k_{fl}}$ rate constant for excited PE, PC, APC, PSII and red states of 0.86/ns has been omitted for clarity

In a simultaneous target analysis of the four data sets collected with 400 and 550 nm excitation and two different time ranges, we linked all SAS, except for the PE SAS which were allowed to differ below 653 nm because of the attenuation on the blue side by the cutoff filter used with 550 nm excitation. An important distinction between PSI and PSII is the rate of trapping. In *Synechococcus* WH 7803 PSI the major trapping lifetime in vitro was 18 ps, with some trapping and equilibration with a redshifted Chl in 7.5 ps (van Stokkum et al. 2013). Thus, the biexponential decay of the PSI emission can be described with two compartments for bulk and redshifted Chl that equilibrate. Trapping is modelled with a decay rate of 80/ns from the bulk Chl. In these experiments, one cannot distinguish between different models for EET and trapping in PSII (Holzwarth et al. 2006; Raszewski and Renger 2008). The biexponential decay of the PSII dimer emission (Tian et al. 2013a) has been described by an equilibrium of the Chl *a* compartment with a radical pair (RP) compartment. The rate from the PSII Chl *a* to RP was estimated to be 14/ns. This large difference in the trapping dynamics between PSI and PSII allows estimation of the rates of EET from PB to PSI or PSII. When after 550 nm excitation and EET the Chl emission ultimately decays with a rate much faster than 14/ns, it can be concluded that an appreciable amount of the PB excitations is quenched by trapping in PSI. The EET rate to PSII was estimated to be 50/ns, which is the same as the value estimated by Acuña et al. (2017) in a target analysis of whole cells of a PSI-deficient mutant of *Synechocystis* sp. PCC 6803. The estimated relative precision of each estimated rate constant was 20%. The most striking finding of the estimated rates in Fig. 4 is that the EET rate to PSI is larger than that to PSII. This is in agreement with the efficient EET (estimated quantum efficiency up to 0.89) from PB to PSI determined in *Synechococcus* sp. PCC 7002 (Dong et al. 2009).

The EET rate between PE and PC645 is heterogeneous. The fast fraction (68/ns) is 73 or 62% with 550 or 400 nm excitation. The remainder (27 or 38%) transfers with a slow rate of 11 or 9/ns with 550 or 400 nm excitation. The fast fraction can be attributed to the PE pigments that are most close to the PC645 pigments, which are located in the PC hexamer (Ong and Glazer 1987) and in the neighbouring PEI hexamer. The slow fraction can be attributed to the PE pigments more distal to the core. The fast rate is similar to the intrahexamer EET rate of 69/ns, whereas the slow rate is very close to the effective rod to core EET rate of 12/ns that were both estimated in *Synechocystis* (Acuña et al. 2017; van Stokkum et al. 2017). The estimated EET rates are consistent with calculations of Förster EET rates in rods (Xie et al. 2002).

**Fig. 5:**
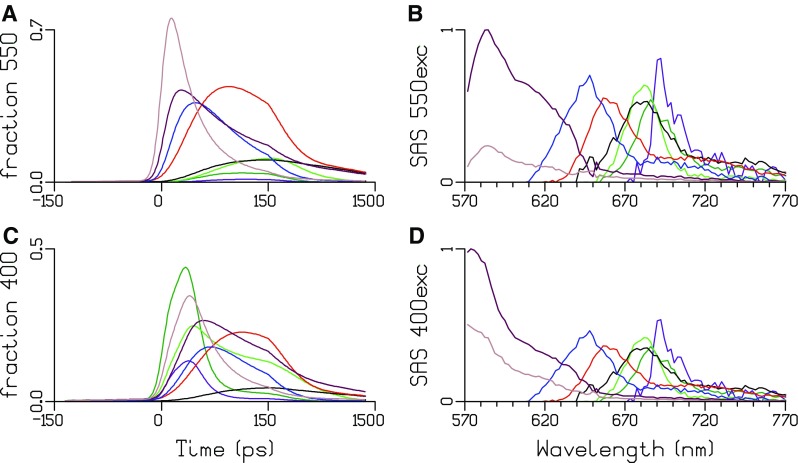
Total concentrations and SAS estimated after 550 (**a, b**) or 400 (**c, d**) nm exc. Key: PEII (brown), PEI (maroon), PC645 (blue), APC660 (red), APC680 (black), PSII Chl *a* (green), PSI Chl *a* (dark green) and PSI “Red Chl” (purple)

In panels A and C of Fig. 5, the total concentration is plotted. For each species, the total concentration is the sum of all excited state populations in the compartments with the spectrum of that species. Note that the initial population of PSI and PSII Chl *a* greatly increases with 400 nm excitation (Fig. 5c), which is absorbed well due to the Soret band of Chl *a*. The percentages excitation of each pigment type are collated in Table 4 and are consistent with the properties of the six types of pigments. Note that APC680 and PSI “Red Chl” are not in Table 4, since the amount of these excited pigments is very small compared to APC660 and bulk PSI Chl *a*. The time zero spectrum is the weighted sum of the SAS of all the excited pigments. Thus, the shape of the SAS of the fastest decaying species, PEI, is the most sensitive to the relative absorption parameters. The parameters of Table 4 that led to the acceptable SAS of Fig. 5 were thus determined iteratively.

**Table 4:** Percentage excitation of each pigment type with the different excitation wavelengths at RT

	400 exc (%)	550 exc (%)
PEI	3.5	16.3
PEII	21.1	79.7
PC645	1.5	1.9
APC660	1.1	2.0
PSI	61.7	
PSII	11.0	

The estimated SAS depicted in Fig. 5d are consistent with the properties of the eight types of pigments. The maxima of the emission are at < 570, 574, 646, 658, 663, 684, 682, 686, and 690 nm for, respectively, PEII (brown), PEI (maroon), PC645 (blue), APC660 (red), APC680 (black), PSII Chl *a* (green), PSI Chl *a* (dark green) and PSI “Red Chl” (purple). The fit quality of the target analysis is good, cf. Figure S1 and Figure S2. Taking into account the large overlap of the SAS, in particular from 640 to 700 nm, it is remarkable that the SAS have been so nicely resolved. Thus, the simple energy funnel type of kinetic scheme of Fig. 4 allows to resolve the complete EET dynamics. Note that all estimated rate constants represent effective rates, and when more would be known about the structure, it would be possible to infer e.g. the EET rates between the APC660 pigments within and between the three core cylinders (van Stokkum et al. 2017).

The estimates for the PSI equilibration are somewhat faster than the rates estimated in vitro (van Stokkum et al. 2013), whereas the estimated trapping rates are very similar in vivo and in vitro, i.e. 80 and 85/ns. The SAS of the “Red Chl” in PSI is shifted by only 4 nm relative to the bulk (690 vs. 686 nm, purple vs. dark green in Fig. 5). In agreement with the in vitro results (van Stokkum et al. 2013), the equilibrium favours the bulk Chl *a* (Fig. 4), which is in contrast to most other cyanobacteria where the “Red Chl” in PSI emits at 708–740 nm (Gobets et al. 2001).

### Measurements at 77 K

**Fig. 6:**
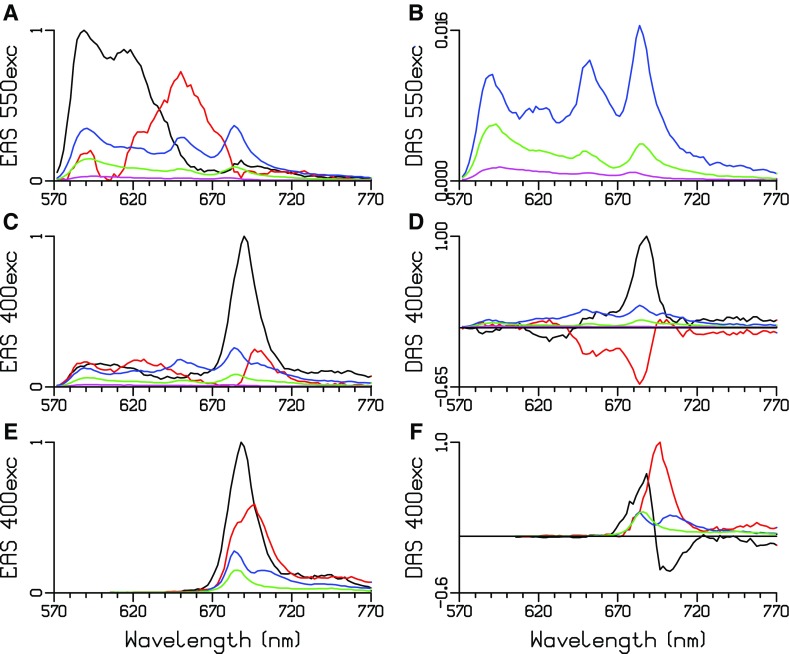
Estimated EAS and DAS at 77 K of whole cells after 550 (**a, b**) or 400 (**c, d**) nm excitation, and of “PSI” (**e, f**) after 400 nm excitation. Estimated lifetimes are collated in Table 5

At 77 K, a global analysis of the whole cells requires five lifetimes with 550 or 400 nm excitation, cf. Fig. 6a–d and Table 5. All EAS (and DAS) are attenuated on the blue side, because a cutoff filter had to be used to suppress the scattered excitation light. The emission below 600 nm can be attributed to PEI and PEII. The PE decay is again strongly multiexponential. With 550 nm excitation, the DAS of the three longest lifetimes below 600 nm indicate slow EET with 98 and 470 ps (blue and green DAS in Fig. 6b). In the final DAS of ≈ 3 ns (magenta in Fig. 6b), the peak at ≈ 590 nm can be attributed to a small fraction of non-transferring PE, whereas the peak at ≈ 677 nm can be interpreted as a small fraction of non-transferring PB. The first two lifetimes with 550 nm excitation are both almost 23 ps; thus, their DAS are compensating and cannot be plotted. A clear redshift of the emission is visible going from the black to the red EAS (Fig. 6a), indicating EET. With 400 nm excitation, in the Chl *a* emission region (670–700 nm), PSI equilibrates and partly decays with 11 ps (black EAS and DAS in Fig. 6c, d). With both excitation wavelengths, the green DAS (≈ 400 ps) shows three peaks (Fig. 6b, d). The peak at ≈ 685 nm can be attributed to decay of low energy states in the photosystems. The peak at ≈ 685 nm of the blue DAS (98 or 64 ps) can be attributed to trapping in the photosystems. The peak at ≈ 650 nm suggests the presence of slowly transferring rods. A target analysis of these 77 K whole cell data (Fig. 6a–d) is a subject of further research.

**Table 5:** Estimated lifetimes at 77 K (in ps) of whole cells after 550 or 400 nm excitation and of “PSI”

Sample	${\lambda _{'{\text{exc}}}}$ (nm)	τ1 (black)	τ2 (red)	τ3 (blue)	τ4 (green)	τ5 (magenta)
Cell	550	22.6	22.9	98	470	2980
Cell	400	11	21	64	317	3420
“PSI”	400	10	31	140	1935	

A global analysis of a “PSI” sample (with 400 nm excitation) requires four lifetimes, cf. Fig. 6e, f. The shortest lifetime of 10 ps can be attributed to equilibration (black DAS in Fig. 6f). Next, 31 ps is the main trapping lifetime in PSI (red DAS in Fig. 6f). Both the blue and green DAS (Fig. 6f) show a peak at ≈ 685 nm that can be attributed to decay in a fraction of PSII that is present in this “PSI” sample. The peak at ≈ 702 nm in the blue DAS (Fig. 6f) can be attributed to the decay of low energy states in PSI. These complicated DAS can be further interpreted with the help of a target analysis.

**Fig. 7:**
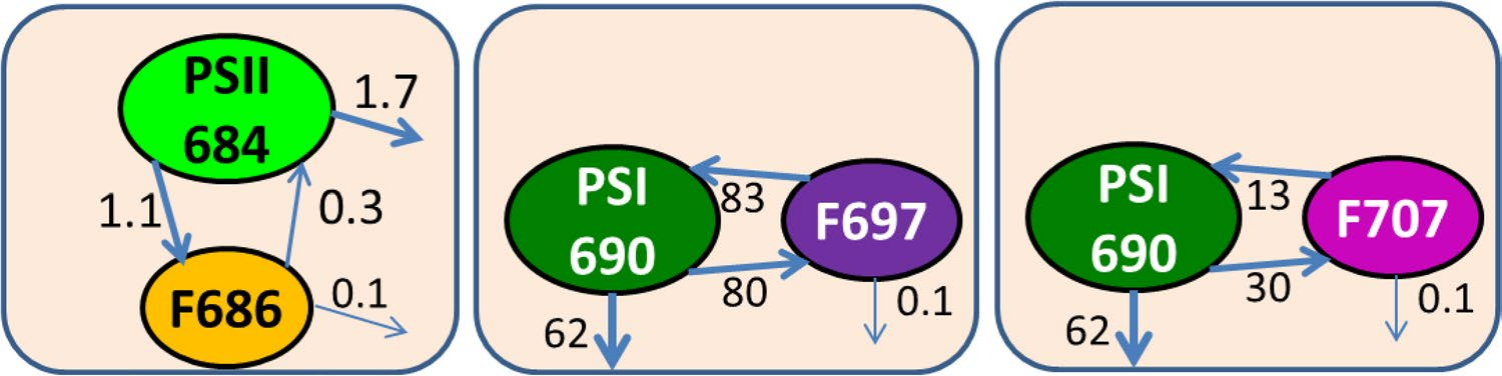
Functional compartmental model of PSI and PSII at 77 K. All microscopic rate constants are in 1/ns. Further explanation is provided in the text

Several models exist for PSI and PSII at 77 K (Snellenburg et al. 2013, 2017; Tian et al. 2013a) which consist of compartments for bulk Chl *a* in equilibrium with low energy states present in the photosystems. In PSII, at least one, and often two, low energy states are needed. In PSI, the number of low energy states is species dependent. The bulk Chl *a* decays by trapping (charge separation, photochemical quenching) in the PSI RC and by some quenching process that probably involves formation of a radical pair in the closed PSII RC. The kinetic scheme of Fig. 7 can describe the data of the “PSI” sample, which contained a fraction of PSII. PSII bulk Chl *a* is in equilibrium with one F686 compartment that represents all the redshifted Chl *a* in the CP43 and CP47 core antenna complexes. This equilibrium decays very slowly (4 ns). At 77 K, the heterogeneity of the Red Chl in PSI has to be taken into account (Gobets and van Grondelle 2001). This is modelled by two equilibria between bulk Chl *a* and a redshifted Chl *a* compartment. These redshifted Chl *a* compartments are denoted by their emission maximum F697 and F707. Crucial for resolving the five PS SAS in the target analysis are the spectral constraints. The shape of the vibrational band of the two PSII SAS has been assumed to be equal above 690 nm. The SAS of PSI F707 has been assumed to be zero below 695 nm. In addition, to resolve the equilibria, an equal SAS area constraint was instrumental (Snellenburg et al. 2013). The fit quality of the target analysis is excellent (Figure S3).

**Fig. 8:**
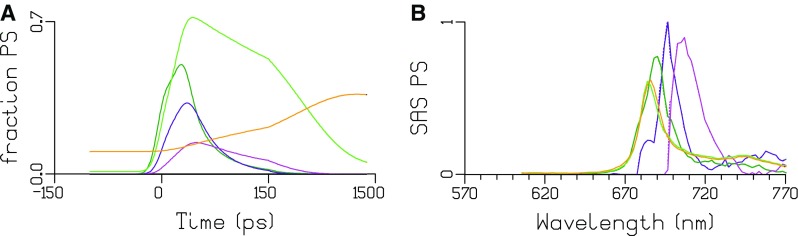
Total concentrations (**a**) and SAS (**b**) estimated after 400 nm exc of the “PSI” sample. Key: PSII bulk Chl *a* (green), PSII F686 (orange), PSI bulk Chl *a* (dark green), PSI F697 (purple) and PSI F707 (magenta)

The populations of the PS compartments are depicted in Fig. 8a, and the amplitude matrix that summarizes their dynamics is given in Table 6. In (0.54/0.75 =)72% of PSI, the bulk Chl *a* (dark green) and PSI F697 (purple) equilibrate with a lifetime of 5 ps, and this equilibrium decays by trapping with a lifetime of 39 ps. In the remaining 28% of PSI, the bulk Chl *a* (dark green) and PSI F707 (magenta) equilibrate with a lifetime of 10 ps, and this equilibrium decays by delayed trapping with a lifetime of 121 ps. Thus, the dominant trapping lifetime in PSI is 39 ps (57%), and minor trapping lifetimes are 5 (15%), 10 (17%) and 121 ps (11%). PSII bulk Chl *a* (green) and PSII F686 (orange) equilibrate with a lifetime of 341 ps. The PSII emission decays with lifetimes of 341 ps (54%) and 4.4 ns (46%).

**Table 6:**
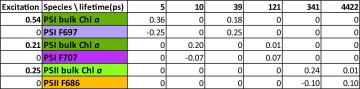
Amplitude matrix of the “PS1” sample at 77 K with 400 nm excitation

Color code of the species and estimated microscopic rates are given in Fig. 7. Further explanation is provided in the text

In PSI cores of other cyanobacteria (Gobets et al. 2001), maxima at 708 and 719 nm were resolved (at RT) in *Synechococcus elongatus* and *Synechocystis* sp. PCC 6803, and additionally at 740 nm in the trimeric core of *Spirulina platensis*. At RT, these states have a lower free energy than P700, in contrast to the “Red Chl” state in *Synechococcus* WH 7803 that peaked at 690 nm (Fig. 5). At 77 K, the inhomogeneity of this “Red Chl” can be modelled by two populations F697 (72%) and F707 (28%). F697 can be trapped faster than F707 because the latter has a lower free energy, and the EET to bulk Chl *a* is smaller. Thus among cyanobacteria, the PSI “Red Chl” in *Synechococcus* WH 7803, that peak at 697 and 707 nm at 77 K, are the least redshifted PSI “Red Chl”.

## Electronic supplementary material

Below is the link to the electronic supplementary material.

Supplementary material 1 (PDF 4702 KB)

## Abbreviations

APC: Allophycocyanin
DAS: Decay-associated spectrum
EAS: Evolution-associated spectrum
EET: Excitation energy transfer
FWHM: Full width at half maximum
IRF: Instrument response function
PB: Phycobilisome
PC: Phycocyanin
PE: Phycoerythrin
PS: Photosystem
PU: Phycourobilin
RP: Radical pair
rms: Root mean square
SAS: Species-associated spectrum
SNR: Signal to noise ratio
SVD: Singular value decomposition
TRES: Time-resolved emission spectrum
