# Supplementary material for: Energy transfer and trapping in Synechococcus WH 7803
Source: Photosynth Res. 2017 Oct 13;135(1):115–24. doi: 10.1007/s11120-017-0451-2 (PMC5784009; doi:10.1007/s11120-017-0451-2)
Supplement: Supplementary file 1 — Supplementary material 1 (PDF 4702 KB) [file 11120_2017_451_MOESM1_ESM.pdf]

Supplementary material to

Energy Transfer and Trapping in *Synechococcus* WH 7803

Alonso M. Acuña, Claire Lemaire, Rienk van Grondelle, Bruno Robert, Ivo H.M. van Stokkum

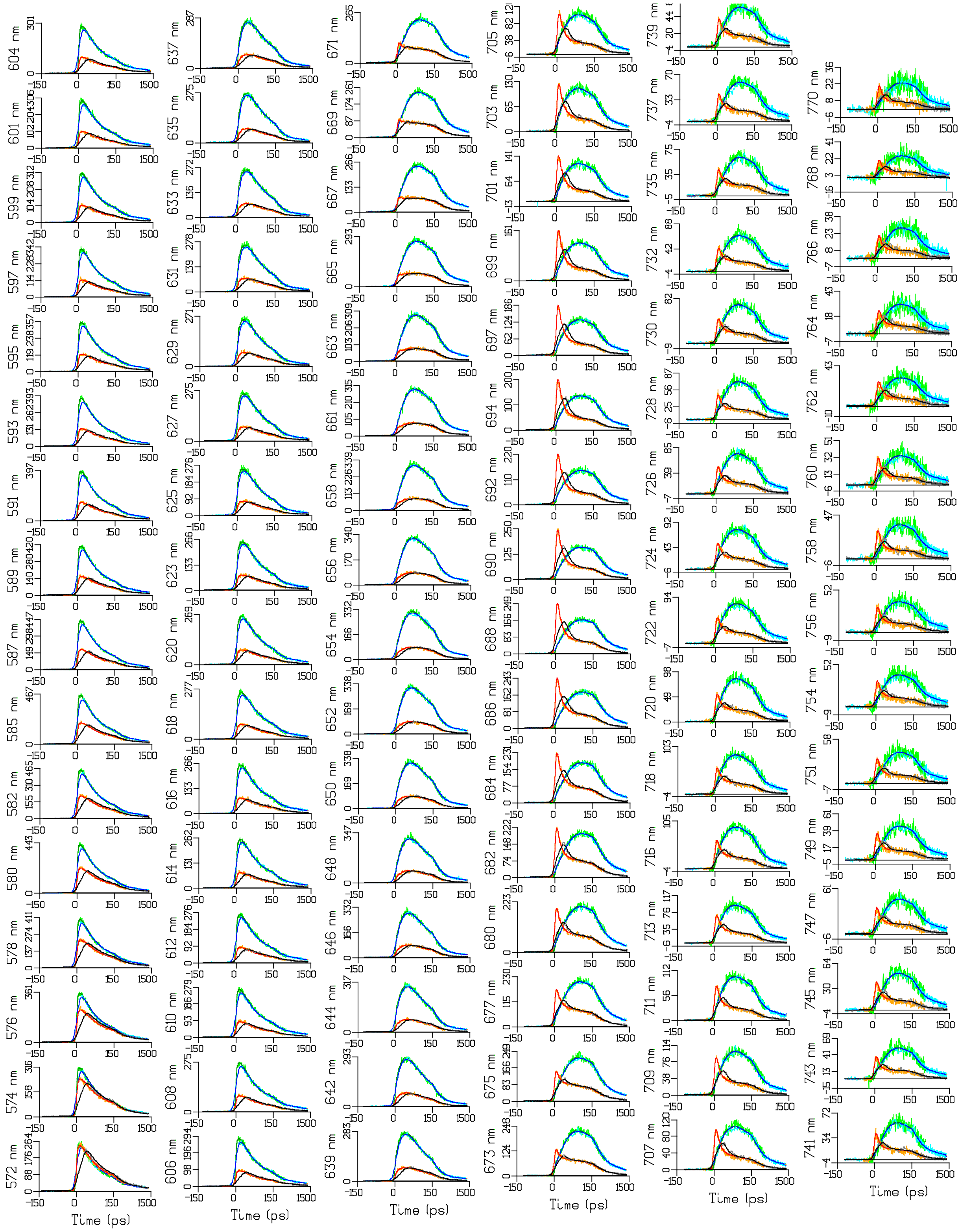

Figure S 1. Emission at 95 wavelengths (indicated in the ordinate label) after 400 or 550 nm excitation at RT. Key: TR4, 400 (grey), TR2, 400 (orange), TR4, 550 (cyan), TR2, 550 (green). Black, red, blue and dark green lines indicate the simultaneous target analysis fit. Note that the time axis is linear until 150 ps and logarithmic thereafter. Note also that each panel is scaled to its maximum. Overall rms error of the fit was 1.54.

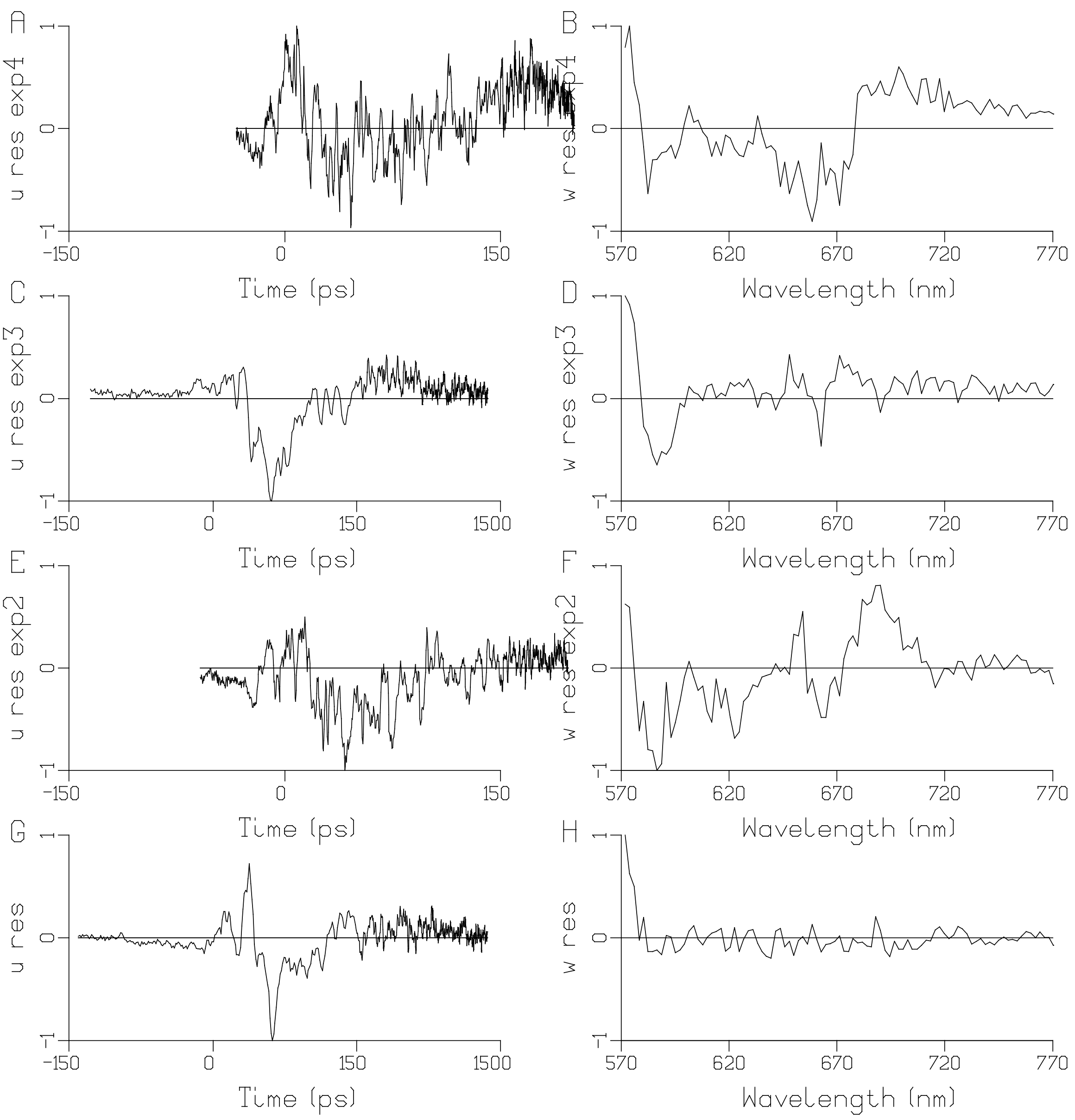

Figure S 2. First left and right singular vectors u_1 (left column) and w_1 (right column) of the residual matrix after a simultaneous target analysis of the 550 and 400 nm excitation data at RT. (A,B) TR2, 550 (green in Figure S 1), (C,D) TR4, 550 (cyan in Figure S 1), (E,F) TR2, 400 (orange in Figure S 1), and (G,H) TR4, 400 (grey in Figure S 1). Note that the time axis is linear until 150 ps and logarithmic thereafter. Most of the structure in the first left singular vectors (A,C,E,G) is straddling time zero, the location of the IRF maximum. This can be attributed to small inaccuracies in the description of the IRF.

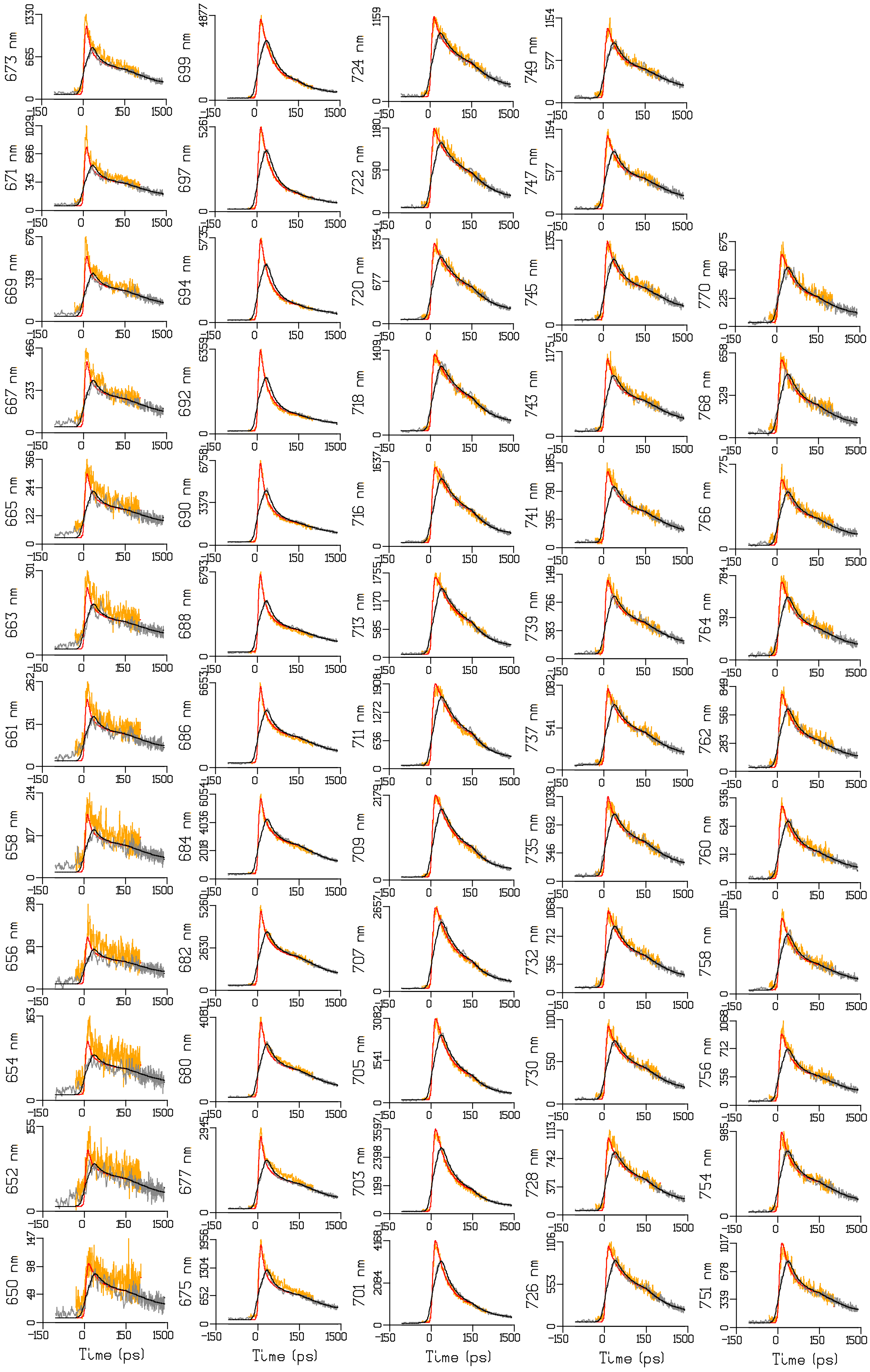

Figure S 3. Emission at 58 wavelengths (indicated in the ordinate label) after 400 nm excitation of “PSI” at 77K. Key: TR4 (grey), TR2 (orange). Black and red lines indicate the simultaneous target analysis fit. Note that the time axis is linear until 150 ps and logarithmic thereafter. Note also that each panel is scaled to its maximum. Overall rms error of the fit was 19.6.
